# Methylmercury Exposure and Health Effects from Rice and Fish Consumption: A Review

**DOI:** 10.3390/ijerph7062666

**Published:** 2010-06-21

**Authors:** Ping Li, Xinbin Feng, Guangle Qiu

**Affiliations:** State Key Laboratory of Environmental Geochemistry, Institute of Geochemistry, Chinese Academy of Sciences, Guiyang, 550002, China; E-Mails: ping_ligyig@163.com (P.L.); qiuguangle@vip.skleg.cn (G.Q.)

**Keywords:** rice, fish, methylmercury exposure, nutrition, health effects

## Abstract

Methylmercury (MeHg) is highly toxic, and its principal target tissue in humans is the nervous system, which has made MeHg intoxication a public health concern for many decades. The general population is primarily exposed to MeHg through consumption of contaminated fish and marine mammals, but recent studies have reported high levels of MeHg in rice and confirmed that in China the main human exposure to MeHg is related to frequent rice consumption in mercury (Hg) polluted areas. This article reviews the progress in the research on MeHg accumulation in rice, human exposure and health effects, and nutrient and co-contaminant interactions. Compared with fish, rice is of poor nutritional quality and lacks specific micronutrients identified as having health benefits (e.g., n-3 long chain polyunsaturated fatty acid, selenium, essential amino acids). The effects of these nutrients on the toxicity of MeHg should be better addressed in future epidemiologic and clinical studies. More emphasis should be given to assessing the health effects of low level MeHg exposure in the long term, with appropriate recommendations, as needed, to reduce MeHg exposure in the rice-eating population.

## 1. The Toxicity of Methylmercury

Methylmercury (MeHg) is a potent toxicant. Since the first reported outbreak of MeHg poisoning in Minamata (Japan) in 1956, the toxicity of MeHg has been researched by scientists worldwide. Numerous reports and review articles discussing this issue have been published, among which we may mention the WHO’s Methylmercury-Environmental Health Criteria 101 [1], the USEPA Mercury (Hg) Study Report to Congress, Volume V: Health Effects of Mercury and Mercury Compounds [2], the Toxicological Profile for Mercury by ATSDR [3], Toxicological Effects of Methylmercury by the US NRC [4], and the reviews by Clarkson [5], Clarkson and Magos [6] and Mergler *et al.* [7]. In this section, which builds on earlier literature and review articles, we briefly describe the current scientific knowledge on human MeHg exposure and the related health effects.

### 1.1. Pathways of Human Exposures

Chemists first synthesized MeHg in the mid-19th century in the form of dimethylmercury [8]. However, nowadays human exposure occurs almost exclusively to MeHg from consumption of fish and marine mammals. MeHg contamination in fish poses a particular challenge to public health, because fish is a highly nutritious food, with known benefits for human health. Moreover, fish are culturally vital for many communities and constitute an important global commodity [7].

MeHg is present as a result of the methylation of inorganic Hg by microorganisms usually present in sediments. It undergoes a remarkable bio-magnification process and accumulates in the fish muscle tissues of long-lived predatory species such as pike in fresh water and sharks in ocean waters. The degree of bio-magnification depends on the location of the fish species in the food chain. For example, in unpolluted ocean water, the herbivorous reef fish may have concentrations of MeHg as low as 0.01 μg/g, whereas sharks may reach levels as high as 4 μg/g. The maximum allowed or recommended levels of MeHg in fish recommended by the Joint FAO/WHO Expert Committee on Food Additives [9] are 1.0 μg/g for predatory fish and 0.5 μg/g for other fishery products and this guideline has been adopted by most countries.

The chemical form of MeHg in fish tissue has recently been identified as attached to the thiol group of the cysteine residues in fish protein [10], which are not removed and destroyed by any cooking or cleaning processes. Although fish are the predominant sources of MeHg in the human diet, a few reports of other sources exist. Rice cultivated in Hg contaminated areas can contain relatively high levels of MeHg [11–15] and the main route of human MeHg exposure there is related to frequent rice consumption [13]. MeHg has also been reported in meats of terrestrial animals [16], probably as a result of the use of contaminated fish meal as a livestock feed [17].

### 1.2. Toxicokinetics

Although most experimental studies on the gastrointestinal absorption of MeHg indicated that nearly 100% of MeHg in fish is absorbed, recently reported animal and human data suggest that there may be substantial variability [18,19]. After the distribution from the blood compartment to all the body tissues is completed, a process that takes about 30 to 40 h, on average about 5% of the absorbed dose remains in the blood compartment [6].

Toxicokinetic (pharmacokinetic) models and physiologically based pharmacokinetic (PBPK) models are applied to estimate internal dose [4]. The basic one compartment model [1,20,21] is a steady state model that is intended to predict concentration in a single compartment only (generally, blood). However, its relative simplicity has allowed it to be used with probabilistic input parameters to obtain estimates of population variability in predictions of blood concentration and intake dose [22]. Nevertheless, there is increasing evidence suggesting that diet and/or metabolic differences may influence MeHg uptake and/or excretion [19].

MeHg has a relatively long half life of approximately 70–80 days in the human body [2]. MeHg is transported out of liver cells into bile as a complex with reduced glutathione using glutathione carriers [23,24]. These transport pathways also play a key role in the elimination of MeHg from the body. In humans, approximately 90% of the absorbed dose of MeHg is excreted in the feces as mercuric Hg [2].

### 1.3. Biological Indicators

Hair and blood Hg concentrations are both considered as good biomarkers of MeHg exposure, although each provides different assessments of exposure [4]. With respect to prenatal exposure to MeHg, both cord blood and maternal hair have been used [6]. Blood gives an estimate of exposure over the most recent one to two half-lives, whereas hair reflects the average exposure over the growth period of the segment [25].

For the population that frequently consumes fish, MeHg is the predominantly found phase of Hg in hair [ranging from 80% to 98% of hair total Hg (THg)] [1,26] and hair THg and blood MeHg are consistently correlated [27]. Generally, Hg in hair is 250 to 300 times more concentrated than that in blood [1,2]. Segmental analyses of hair Hg can provide a useful chronology of exposure over time [25,26,28,29], on a basis of an assumed hair growth rate of 1 cm per month [4]. Recent advances in single strand analysis at micron resolution by laser ablation can provide more information on Hg deposition in hair [30,31]. The Hg levels in toenails and finger nails also were used as biomarkers of MeHg exposure, mostly in the studies of cardiovascular effect of MeHg [32,33].

### 1.4. Health Effects

Since the major adverse effects of MeHg exposure are neurological, we discuss this in detail. For renal, cardiovascular, reproductive, and immune system effects, we briefly summarize the new findings.

#### 1.4.1. Neurological Effects

The brain and nervous system are the primary target tissues for the effects of MeHg in adults and also neonates, infants, and children [1]. MeHg poisoning results in marked distal sensory disturbances, constriction of visual fields, ataxia, dysarthria, auditory disturbances, and tremors [34,35]. The WHO [1] estimated that 5% of MeHg exposed adults would experience some neurologic effects with a blood Hg level of 200 μg/L (corresponding to a hair level of 50 μg/g).

Data on the impact of chronic low dose of MeHg exposures on adult neurological effects have been obtained from studies of fish eating populations in the Amazon Basin, where Hg pollution has from artisanal gold mining in commonplace. Hg related deficits in motor, psychomotor, visual and/or cognitive functions have been reported [36–38].

The Minamata MeHg poisoning episode brought attention to the risks of fetal exposure. Infants exposed to MeHg through the placenta of the exposed mother showed severe cerebral palsy-like symptoms, even when their mothers displayed mild or no manifestations of MeHg poisoning [39]. Mental retardation, cerebellar ataxia, primitive reflexes, dysarthria, and hyperkinesias were all observed [34,40]. It has been estimated the risk of fetal brain damage increases when the Hg concentration in maternal hair exceeds a level of 10–20 μg/g [4].

Most attention has been paid to the three major epidemiological studies on children development from New Zealand [41,42], the Faroe Islands [43,44] and Seychelles [45,46], but the results of the three studies differed. The New Zealand study reported a greater percentage of offspring in a high Hg group (maternal hair Hg levels >6 μg/g) had lower developmental scores compared to offspring from the low Hg groups (<3 μg/g, 3–6 μg/g). In the Faroe Islands, where residents periodically consume whale meat that is high in Hg (with an average of 1.6 μg/g), and the authors also reported higher Hg levels in cord blood associated with lower developmental scores. In the Child Development Study on the Seychelles, where residents consume daily a wide variety of ocean fish that are low in Hg (<0.3 μg/g), the authors reported no association between maternal Hg levels in hair and developmental scores on neurological tests.

#### 1.4.2. Renal Effects

Renal toxicity has rarely been reported following human exposure to MeHg. The only evidence of a renal effect following ingestion of Hg contaminated fish comes from a death certificate review [47]. The number of deaths attributed to nephritic diseases was found to be higher than expected among women in Minamata City with the highest prevalence of Minamata disease.

#### 1.4.3. Cardiovascular Effects

A body of evidence was developed that addresses potential associations between MeHg and a range of cardiovascular effects. These include cardiovascular disease (coronary heart disease, acute myocardial infarction (AMI), ischemic heart disease), blood pressure and hypertension effects, and alterations in heart rate variability [48,49]. There are strong evidences for causal associations with cardiovascular disease, particularly AMI in adult men [32,50–52].

#### 1.4.4. Reproductive Outcomes

The effect of MeHg on the sex ratio of offspring at birth and stillbirth in Minamata City, Japan, in the 1950s and 1960s, showed decreases in male birth in offspring in the overall city population, among fishing families [53,54].

#### 1.4.5. Immune System Effects

Both MeHg and inorganic Hg were shown to produce an autoimmune response, as well as an immunosuppressive effect in several strains of genetically susceptible mice [55–57]. However, data on this issue in general are sparse, and further research is required in this area.

## 2. MeHg in Rice and Risk Assessment

Rice is the second highest produced cereal in the world and the annual production had reached 410 million tons. Asia is by far the biggest rice producer, accounting for 90% of the world’s production and consumption of rice. Rice is the staple food for more than half of the world’s population, contributing not only up to approximately 80% of energy intake in some regions [58], but it can account for a significant proportion of the daily doses of protein and micronutrients [59].

Generally, Hg concentrations in most foodstuffs are below 20 ng/g, and is mainly present in inorganic forms [60], but recent studies, listed in Table 1, have reported high levels of MeHg in the rice from Hg polluted areas. Horvat *et al.* [11] first reported that rice produced in the Hg polluted Qingzhen area and Wanshan Hg mining area in Guizhou Province, China, contained elevated levels of MeHg and the maximum value was up to 144 ng/g. Shi *et al.* [61] investigated MeHg concentrations in 25 rice samples produced in 15 provinces in China, and the MeHg concentrations ranged from 1.9 to 10.5 ng/g, with a average value of 4.5 ng/g.

Qiu *et al.* [12] found elevated high THg concentrations (8.9–570 ng/g, dry weight) in agricultural crops from the Wuchuan Hg mining area, but high MeHg concentrations, which ranged from 4.2 to 18 ng/g, were only observed in rice. The proportion of MeHg to THg in rice was found to be up to 66%. The results indicated that rice produced in the Hg mining areas in Guizhou Province showed high MeHg accumulation ability. Feng *et al.* [13] conducted a study of hair MeHg levels of 98 persons and rice Hg levels from the Wanshan Hg mining area. Rice, the staple food of the local inhabitants showed not only high THg, but also MeHg levels. The geometric mean of MeHg in rice samples collected from three villages in Wanshan were 8.5 ng/g (ranging from 1.9 to 27.6). Pork meat, vegetables, and drinking water samples contained highly elevated THg, but very low levels of MeHg. The significant relationships between the estimated rice MeHg intake and hair MeHg levels (r = 0.65, p < 0.001) confirmed rice indeed was the main route of MeHg exposure for the local residents. This indicated that human MeHg exposure via food consumption is not restricted to fish, but in some cases is related to frequent rice meals. Li *et al.* [14] reported elevated MeHg level in rice from the Wuchuan Hg mining area, which ranged from 3.1 to 13.4 ng/g. The hair MeHg investigation indicated the population in the study area was at a potential risk of MeHg exposure via rice intake.

Qiu *et al.* [15] reported that rice (*Oryza sativa L.*) grown in abandoned Hg mining areas contained MeHg levels of 1.61–174 ng/g in the edible portion and it proved to be 10–100 times higher than other crop plants. The results demonstrate that rice is a MeHg bio-accumulative plant and the main MeHg source for human exposure in the study areas

The elevated MeHg level in rice may be likely linked with a high methylation ability in paddy soils. Seasonal irrigation of paddy field produces anaerobic conditions in surficial soil, which favors *in situ* methylation of inorganic Hg [62–64]. However, the mechanism of the uptake of MeHg in rice tissue is still unclear.

To estimate the daily MeHg intake through rice consumption, we assumed that daily ingestion of rice was 600 g for adults and the body weight is 60 kg. The daily MeHg exposures were expressed as μg/kg/d (μg MeHg/kg (body weight)/d (day)) for the rice eating population and the results were given in Table 1.

The Joint FAO/WHO Expert Committee on Food Additives (JECFA) established a provisional tolerable weekly intake (PTWI) for MeHg to 1.6 μg/kg/week, which is equivalent to 0.23 μg/kg/d [9]. The United States Environmental Protection Agency (USEPA) set the limit of 0.1 μg/kg/d as reference dose (RfD) for MeHg [2]. From the daily exposure levels listed in Table 1, we found that most residents in Hg mining areas exceeded acceptable limit suggested by USEPA and some of them exceeded the JECFA level. Although the MeHg concentrations in rice are much lower than that in fish tissue, rice intake in Hg mining areas may pose a health risk of MeHg exposure to local habitants due to large amount of daily intake. Especially for the sensitive population, such as the newborn and pregnant women, more attention should be paid to this.

## 3. Hair MeHg levels

**Figure 1.**
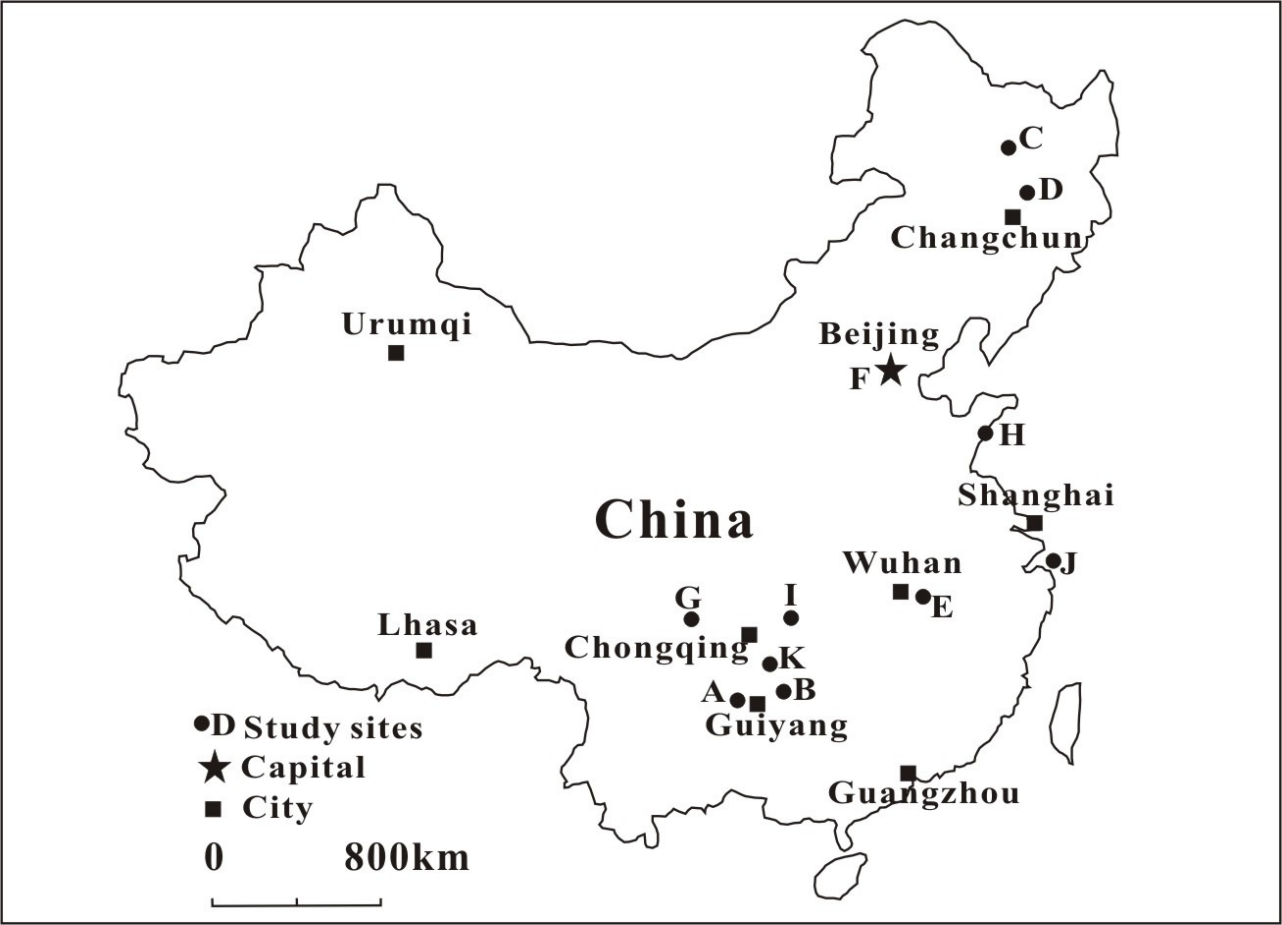
Location of study sites of the fish Hg survey in China.

China is considered as the largest source of anthropogenic Hg emissions, which come mainly from coal combustion and metal smelting [65,66]. The large amount of emitted Hg may become a threat to the local and global environment. Serious Hg pollutions in local environments were found in the vicinity of Hg mining, amalgamation gold mining, chemical plant, metal smelting and coal combustion power plant [67–69]. But the MeHg concentrations in fish tissue in China were very low, even in the heavily Hg contaminated areas, for instance, the Baihua Reservoir, Songhua River, and Di’er Songhua River, which were historically polluted by Hg from chemical plants. The comparisons of fish MeHg level in China were given in Table 2 and the location distribution was shown in Figure 1. Even in the coastal (Qingdao, north China) and island (Zhoushan, east China) areas, the Hg concentrations in fish were also very low.

The low level of Hg in fish in China may be due to: (1) the aquatic food chains are generally short, which does not favour the bioaccumulation of MeHg in fish; (2) most of the fish (mainly herbivorous or omnivorous fish) were produced by aquaculture, where the fish are very young and the growth rate is very fast [70].

Low fish Hg concentration has logically resulted in low hair Hg levels in the fish eating population in China. Thus low average hair THg concentrations (<1 μg/g) were reported in fish eating population in five coastal sites (Shanghai, Ningbo, Dalian, Xiamen, and Zhoushan) in eastern China [81], Changchun City, northeastern China [82] and Di’er Songhua River, northeastern China [83].

**Figure 2.**
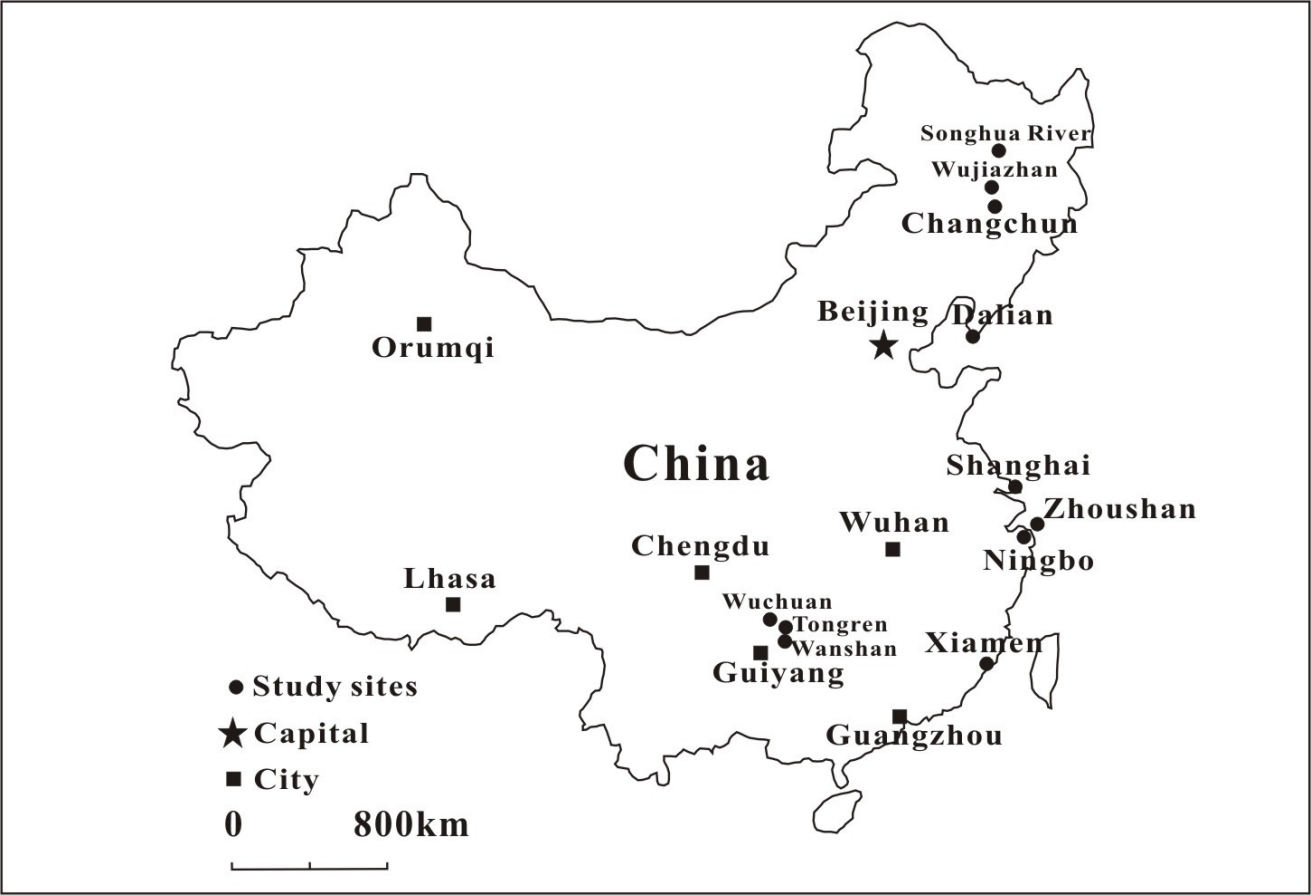
Location of study sites of hair Hg investigation in China.

The comparisons of hair Hg level in the fish and rice eating population over the world were given in Table 3 and the location distribution of the studies in China are shown in Figure 2. Among the studies in China, slightly higher average hair Hg levels were observed in fish eating populations in Zhoushan [79,88] and Songhua River, northeastern China [89]. Even so, the hair Hg levels were much lower than those seen from the studies in the Amazon, New Zealand, Faroe Islands and Seychelles.

Higher MeHg hair levels were reported for the residents in the Hg mining areas of Guizhou Province, despite significantly lower fish consumption (220–310 g/y) [13] and the populations in the Hg mining areas were exposed to MeHg primarily through rice ingestion (81–99%) [13]. The mean hair MeHg concentrations were 2.80, 1.30 and 1.50 μg/g for the residents in three villages in the Wanshan Hg mining area, respectively [13]. The mean hair MeHg concentrations were 4.26 μg/g (1.87–10.6 μg/g) and 4.55 μg/g (2.29–9.55 μg/g) for the population in two artisanal Hg mining area in Tongren, respectively [90]. The mean hair MeHg concentration for the population in the Wuchuan Hg mining area was 1.38 μg/g (with range of 0.45–5.89 μg/g) [14]. From these results, we found that the populations in Hg mining area were at a potential risk of exposure to MeHg. The health risks of MeHg exposure through rice eating are not only limited to Hg mining areas in Guizhou Province, and may be important in other provinces in China [61].

## 4. The Impacts of Nutrition and Co-Contaminants on MeHg Intoxication

### 4.1. Selenium

Selenium (Se) is an essential nutrient that is required for normal function of enzymes that protect brain and endocrine tissues from oxidative damage [91]. There are data indicating that selenium co-administered with MeHg can form selenium-MeHg complexes [92]. A common association between the metabolism of selenium and MeHg is the thiolcontaining peptide glutathione (GSH). Chen *et al.* [93] found Hg exposure increased both selenoprotein and glutathione peroxidase (GSH-Px) in Hg miners compared with the control group. Selenoproteins may bind more Hg through the selenol group and their antioxidative properties help eliminate the reactive oxygen species induced by Hg. A number of studies have examined the interaction of selenium and Hg in various systems, and these have recently been reviewed by Chapman and Chan [18]. But no epidemiologic studies, however, have shown a correlation between Se intakes and the occurrence or absence of symptoms for MeHg intoxication [18,94]. Inconsistencies were also observed in the protective effects seen in animal studies [95]. Raymond *et al.* [96] used the index of Selenium Health Benefit Value (SE-HBV) to integrate mercury-selenium relationships and to better understand the differences between the findings of different maternal Hg exposure studies. But the role of Se remains to be confirmed in MeHg intoxication.

According to a WHO report [97], the typical Se content of foods varies as follows: organ meats and seafood, 0.4 to 1.5 μg/g; muscle meats, 0.1 to 0.4 μg/g; most agricultural crops, 0.1 to 0.2 μg/g; fruits and vegetables, <0.1 μg/g. Brazil nuts are the richest source of food Se, but the content is very variable, ranging from 0.03 to 512 μg/g fresh weight [98].

Selenium is known to bio-accumulate in fish, so exposure to MeHg from fish consumption may be associated with exposure to increased levels of Se. In Japan, fish was the greatest Se contributor (up to 60% of daily total intake) rather than the staple foods (rice and vegetables) [99]. Literature on Se content in fish from different locations ranged between 62.7 and 507 ng/g in Greece [100], 120–632 ng/g in Australia [101], 126 to 502 ng/g in the USA [102], 152–788 ng/g in Korea [103], 195 to 512 ng/g in New Zealand [104], and 100–1,210 ng/g in Brazil [105].

For the less fish eating population, cereals may be the dominant Se source, especially for the global malnourished population. Williams *et al.* [106] evaluated Se distributions for the global rice. U.S. and India rice were found to be the most enriched, with the mean of 180 and 157 ng/g, respectively. Egyptian rice was the lowest with mean values of 9 ng/g. As the biggest rice production country, China possessed the greatest variation of 1,368 ng/g, but the mean value was 88 ng/g.

**Figure 3.**
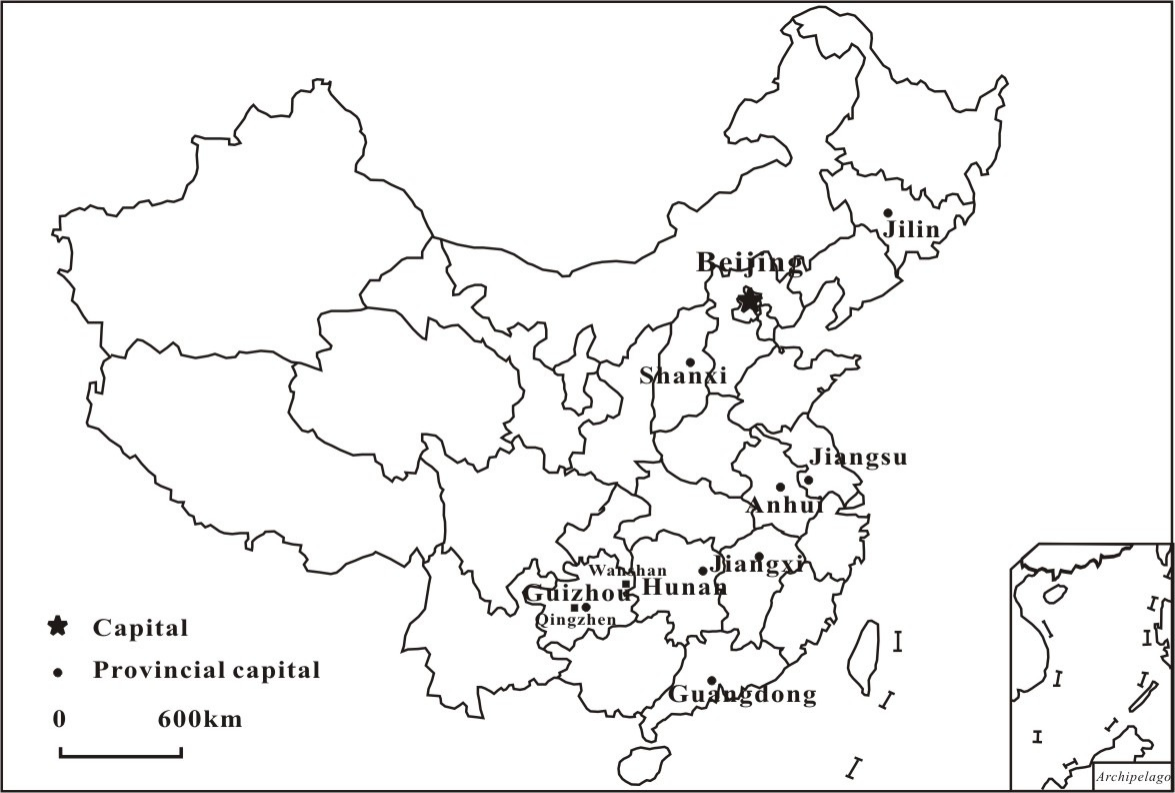
Location of sample sites of Se concentrations in rice from different provinces in China.

The comparison of selenium concentrations in rice from China was given in Table 4 and the location distribution is shown in Figure 3. The selenium concentrations of the regular rice in China, were <0.06 μg/g, some being <0.02 μg/g, regarded as seriously low selenium rice. Rice is the staple food for inhabitants in China. However, the contribution to selenium intake for inhabitants from regular rice products was estimated to be 7.5–12.5 μg of Se per person per day if the mean consumption of an adult was 300–500 g of rice product. Because cereal selenium was the main selenium source in the Chinese diet, lower Se in rice products may be responsible for the Se deficiency status of inhabitants in China.

The soil with abnormally high selenium contents mostly resulted from the parent rocks were found only in Enshi in Hubei Province, Ziyang in Shaanxi Province and North of Guizhou Province [114,115]. An endemic disease caused by selenium intoxication was discovered in 1961 in parts of the population of Enshi City, Hubei Province, China [116]. Actually, there are many Se-deficient areas in China and the low Se soils mainly distribute in a broad belt in the middle of the country, running from the northeast to the southwest of China. The diseases-Keshan disease and Kashin-Beck disease, which always occur in the area with low selenium eco-environment, have been found in 329 counties of 16 provinces and in 335 counties of 15 provinces in China, respectively [114]. Thus, the residents in Hg polluted area may be exposed to MeHg through rice consumption, and are in Se deficiency status.

### 4.2. n-3 Long Chain Polyunsaturated Fatty Acids

For the low level of maternal and infants’ exposure to MeHg, the associated health effects with MeHg in fish is unknown. Results differed between the three largest epidemiologic studies, which were conducted in New Zealand [41,42], the Faroe Islands [43,44], and the Republic of Seychelles [45,46,85,117]. The Faroe study consistently observed significant neurodevelopmental deficits at birth and into the early school years, even when children whose mother’s hair Hg levels above 10 μg/g were excluded [118]. But in the initial studies of the Seychelles cohort, no evident effects were observed [45,46]. Recent reports found significant adverse association between prenatal MeHg and PDI at 30 months of age and indicated that the beneficial effects of n-3 long chain polyunsaturated fatty acid (n-3 LCPUFA) can obscure the determination of adverse effects of prenatal MeHg exposure in longitudinal observational studies [119]. The n-3 LCPUFA is a nutrient in fish, for instance, eicosapentaenoic acid (EPA; C20:5 n-3) and docosahexaenoic acid (DHA; C22:6 n-3) have been increasingly identified as having health benefits [120].

DHA has been associated with a number of beneficial effects on neurocognitive and ocular function. These associations include increased visual acuity in newborns [121], better scores on neurodevelopmental test batteries [122–125], and prevention of a number of neuropsychiatric disorders in adults [126,127].

Fish and seafood, meat, and eggs are three main food sources contribute to n-3 LCPUFA intake, with fish and seafood being the major contributor [128]. Meyer *et al.* [128] ascertained the dietary intakes and food sources of individual n-6 and n-3 LCPUFA in the 1995 Australian National Nutrition Survey. As expected, seafood was the main source of n-3 LCPUFA, contributing 71%, while meat and eggs contributed 20 and 6%, respectively. Sakamoto *et al.* [129] found a significant correlation between Hg in Red Blood Cell and plasma DHA in fetus (r = 0.35, p < 0.01) and the results confirmed that both MeHg and DHA which originated from fish consumption and transferred from maternal to fetal circulation [129].

There is increasing evidence from human studies confirming the protective effects of n-3 LCPUFA on the intoxication of MeHg from fish consumption. Guallar *et al.* [32] evaluated the joint association of Hg levels in toenail clippings and DHA levels in adipose tissue with the risk of a first myocardial infarction among men. The results indicated that the toenail Hg level was directly associated with the risk of myocardial infarction, and the adipose-tissue DHA level was inversely associated with the risk. High Hg content may diminish the cardioprotective effect of fish intake. Oken *et al.* [124] studied a U.S. cohort with moderate fish intake which suggested that maternal fish consumption during pregnancy may benefit offspring cognition in infancy but that exposure to higher levels of Hg has adverse effects on child cognition. Wennberg *et al.* [130] discussed the relationship between stroke risk and fish intake which seems to be different in men and women. Increased levels of EPA and DHA do not decrease the risk for stroke and there is no association between stroke risk and Hg at these low levels.

While it is important to reduce the amount of MeHg exposure from the consumption of MeHg contaminated fish, it is equally important to consume fish in order to get enough n-3 LCPUFA. Balancing the risks and benefits of fish consumption has become an increasingly important goal of fish consumption advisories. Utilizing the data from the National Health and Nutrition Examination Survey (NHANES) for survey years 1999–2002, Mahaffey *et al.* [131] estimated the consumption of MeHg and n-3 LCPUFA from fish in the U.S. Significant association was found between estimated 30-day intake from fish consumption for n-3 LCPUFA and MeHg (r = 0.66). Salmon followed by shrimp are the principal sources of n-3 LCPUFA and are lesser sources of MeHg. Tsuchiya *et al.* [132] examined the intake of EPA and DHA in the Korean and Japanese communities. Intakes of these lipids were used as surrogates to characterize the beneficial effect from fish consumption, and the intake of Hg was used to establish the risk from consumption. The Results suggested that both nutritional elements and contaminant concerns need to be quantitatively incorporated into fish consumption guidelines. Ginsberg and Toal [120] provided an integrated analysis for MeHg and n-3 LCPUFA, which use dose-response relationships on common end points and evaluate the net effect on a species-by-species basis. Rice contains very low concentrations of EPA and DHA, and cereal-based products and dishes only contributed 2.55% of total source [128]. MeHg exposure from rice consumption and disparities in n-3 LCPUFA for the rice eating population may mean serious harm due to MeHg exposure in the same exposure level.

### 4.3. Protein

The type and amount of protein consumed might affect the uptake and distribution of Hg in the body. Low protein intakes have been associated with increased Hg in the brain of the mouse [133,134]. Adequate protein intake prolongs survival after oral doses of MeHg [133–136]. Specific amino acids such as cysteine may detoxify MeHg by preventing inhibition of enzymes such as carnitine acyltransferase [137]. However, cysteine can act as a carrier for MeHg across the blood-brain barrier and thus alter Hg distribution by increasing Hg levels in the brain and increasing neurotoxicity [138,139].

Few studies have examined the effects of malnutrition on the metabolism and toxicity of Hg, but malnutrition in general has a deleterious effect. Inadequate protein increased MeHg induced mortality in mice [134], and methionine deficiency during MeHgCl exposure caused an increase in serum prostaglandins [140].

Generally, the contents of protein were 15–25% in fish [141,142] and it is easily digested by human body. Fish proteins are of very high quality, and contain sufficient amounts of all the essential amino acids required by the body for growth and maintenance of lean muscle tissue. The proteins in fish, as well as similar foods of animal origin, make up complete protein sources in many people’s diets around the world. Generally, the contents of protein were about 8% in rice [143]. Rice proteins are of low quality, because they don’t contain all the essential amino acids. Moreover, rice proteins have fiber membrane around and they are more difficult to absorb by human body compared with animal proteins. Therefore, the effects of protein to the toxicity of MeHg in rice should be of more concern.

### 4.4. Other Nutrients

It is well known that fish are also rich in a number of micronutrients such as choline, iodine and iron. All these nutrients are proven to enhance neurodevelopment [122,144–147]. For that reason, the long term goal needs to be a reduction in the concentrations of MeHg in fish, rather than a replacement of fish in the diet by other foods. Unlike the cohort that consumes fish tissue regularly, populations that depend on rice as a staple food may be in poor nutrition and lack of specific micronutrients (e.g., iron, folate, iodine, zinc, choline). Despite overall improved nutrition in China, disparities in nutritional status between rural and urban pregnant are still common [148]. More research works are needed to reveal the nutrition condition in the rice eating population and its effects on the toxicity of MeHg.

### 4.5. Co-Contaminants

Except MeHg, fish and marine mammals may also accumulate other halogenated organics, such as polychlorinated biphenyls (PCB), dioxins, and related compounds, which share some similarities to those observed for MeHg [149]. This can potentially present difficulties and uncertainty in evaluating causality and in constructing MeHg specific dose-response relations and adverse effects. In the Faroe Islands studies, both MeHg and PCBs appear to jointly affect some developmental endpoints. However, although MeHg appeared to enhance the PCB-attributable effects, the PCBs appeared to make a relatively minor contribution to the MeHg specific effects [149,150]. Contradictory findings were observed in a similar study in New York State [151], of which elevated PCB exposure appeared to potentiate MeHg effects. More work remains to be done on the joint effects of MeHg and halogenated organics, as well as other heavy metals (e.g., lead, cadmium, arsenic) that may also be accumulate in fish [152].

Hg mining areas in Guizhou are one of the least developed regions in the province, and the contamination of persistent organic pollutants (POPs) to the local environment is not a concern. Therefore, it will be much easier to determine dose-response relations and quantify health impacts of MeHg exposure without the presence of POPs exposure in Hg mining areas in Guizhou.

In the Hg mining areas, local population may be exposed to Hg vapor through inhalation, which is not only restricted to artisanal Hg mining workers, but also occurs for the general population [13,14,90,153]. It is known that elemental Hg vapor can cross the placenta and accumulate in fetal tissue [154–156], and animal data suggest that elemental Hg has the potential to cause adverse neurologic developmental effects [157,158]. Both elemental Hg and MeHg are metabolized in the brain to the inorganic Hg form [4]. It is unclear whether the ultimate neurodevelopmental toxicant at the cellular level is MeHg itself, the inorganic Hg that is its metabolite, free radicals generated in the conversion to the inorganic species, or some combination of these. If the ultimate toxic form of MeHg is indeed its inorganic Hg metabolite, it suggests that the dose of inorganic Hg to the brain from elemental Hg exposure and MeHg might be cumulative [4]. Co-exposure to Hg vapor and MeHg show an additive effect on the developing central nervous system from animal data [157,158]. However, more research is needed to indentify the biomarker of Hg vapor and MeHg exposure in the human blood and address their contributions to the neurological impairments.

## 5. Conclusions and Future Perspectives

Generally, fish consumption is considered as the primary pathway for human MeHg exposures; however, recent research has revealed that MeHg exposure also occurs through frequent rice intake in Hg polluted areas. Rice can accumulate MeHg to 100 ng/g in its edible portion, which is proven to be 10–100 times higher than other agricultural crops in Hg polluted area. The estimation of daily MeHg intake through rice consumption and hair MeHg investigation confirmed the potential risk of MeHg exposure to local population. Rice does not contain a lots of beneficial micronutrients associated with fish (e.g., n-3 LCPUFA, selenium, the essential amino acids), which means more harmful effects in the same exposure level. Meanwhile, it can provide the best opportunity to quantify dose-response relationship and health impacts for MeHg exposure without these confounding factors. Rice is a staple food for more than half of the world’s population, mostly in developing countries; therefore, it is urgent and critical to evaluate the potential health risks of this emerging issue *versus* MeHg exposure through fish consumption.

There are so many uncertainties and critical factors affecting the MeHg toxicity on MeHg exposure from rice consumption. More studies are needed in these important areas. First, the study areas should not be confined to the Hg mining areas in Guizhou Province in China, as MeHg exposure may be more widespread in other Hg polluted areas over the world. Second, more studies are needed to clarify the mechanism of MeHg accumulation in rice; therefore, control technologies must be developed to prevent MeHg accumulation in the rice grain. Third, the effects of key nutrition (e.g., n-3 LCPUFA, selenium, essential amino acids) on the toxicity of MeHg should be addressed by the comparison with fish consumption. Fourth, the dose-response relationship of MeHg exposure through rice consumption is needed to quantify without so many confusing factor and co-contaminants. Finally, the continuous epidemiologic studies should be conducted to evaluate the health impacts of low level MeHg exposure on the neurological development of offspring.

## Figures and Tables

**Table 1.** Hg concentrations in the rice from Guizhou Province and other regions in China.

**Location**	**MeHg (ng·g^−1^)**	**THg (ng·g^−1^)**	**MeHg/THg (%)**	**Daily MeHg intake (μg·g^−1^·d^−1^)**	**Ref.**
Qingzhen Hg polluted area	0.71–28	2.53–33.5	28.1–83.7	0.005–0.19	[11]
Wanshan Hg mining area	8.03–144	11.1–569	5.46–72.6	0.05–0.96	[11]
	1.9–27.6	4.9–214.7	2.4–75.1	0.01–0.21	[13]
	1.61–174	10.3–1120	1.4–93	0.016–1.74	[15]
Wuchuan Hg mining area	4.2–18	9.1–570	2–66	0.04–0.18	[12]
	3.1–13.4	6.0–113	6.0–83.6	0.03–0.12	[14]
Fifteen Chinese provinces	1.9–10.5	6.3–39.3	7–44	0.02–0.105	[61]

**Table 2.** The comparison of MeHg level in fish from different sites of China/μg·g^−1^.

**Site**	**Location**	**Functional group**	***n***	**MeHg Mean (Range)**	**Remark**	**Ref.**
A	Baihua Reservoir, Guizhou	2, 3	64	0.011 (0.003–0.039)	Historically polluted by chemical plant	[70]
B	Wanshan Hg mining area, Guizhou	3	12	0.060 (0.024–0.098)	Polluted by Hg mining	[71]
C	Songhua River, Northeastern China	1	20	0.103–0.295*	Historically polluted by chemical plant	[72]
		2	89	0.088 (0.041–0.126)*		
		3	2	0.006*		
D	Di’er Songhua River, Northeastern China	1, 2, 3	186	0.09 (0.002–0.66)*	Historically polluted by chemical plant	[73]
E	Ya-Er Lake, Hubei Province	1, 2	40	0.18 (0.005–0. 64)	Historically polluted by chlor-alkali plant	[74]
F	Beijing	1, 3	32	0.0026 (0.0005–0.0084)	Commercial fish in a market	[75]
G	Chengdu, Sichuan	1	13	0.031 (0–0.117)	Fish collected from pound	[76]
		3	36	0.015 (0.002–0.037)		
H	Qingdao, Shandong	1, 2, 3	102	0.049–0.15*	Commercial freshwater and ocean fish in market	[77]
I	The Three Gorges Reservoir	1, 2	32	0.07–0.201	In the middle part of Yangtze River	[78]
J	Zhoushan Island	1, 2, 3	148	0.18 (0.01–0.59)	Freshwater and ocean fish	[79]
K	Wujiang River, Southwestern China	1, 2	228	0.063 (?–0.58)*	The largest branch on southern bank of Yangtze River	[80]

Functional group: 1 = Carnivorous; 2 = Omnivorous; 3 = Herbivorous

* THg.

**Table 3.** The comparisons of hair Hg levels in fish and rice eating population over the world.

**Location**	**Subject**	**Exposure source**	**Hair Hg level/μg·g^−1^ Mean (Range)**	**Ref.**
New Zealand	Mother-child pairs	Fish	MH 8.3 (6.0–86)	[41,42,84]
Faroe Islands	Pregnant women	Pilot whale	MH 4.3 (with a inter-quartile range of 2.6–7.7)	[43,44]
Seychelles	Mother-infant pairs	Fish	MH 5.9 (0.5–26.7)	[45,46, 85]
Amazon	Indigenous and riparian population	Fish	≈15	[86]
			≈2–3	
	Urban residents			
Nine prefectures in Japan	General population	Fish	Male 2.42 (0.10–29.4)	[87]
			Female 1.37 (0.02–25.8)	
Zhoushan Island, China	Pregnant women	Fish	MH 1.25 (0.93–1.68)	[88]
	General population	Fish	Fathers 3.8 (0.9–9.5)*	[79]
			Mothers 1.8 (0.3–4.1)*	
			Children 1.7 (0.6–4.1)*	
Five coastal cities (Shanghai, Ningbo, Dalian, Xiamen, and Zhoushan) in China	General population	Fish	0.83 (0.04–8.48)	[81]
Changchun, Northeastern China	Urban residents	Fish	0.448 (0.092–10.5)	[82]
Wujiazhan, Di’er Songhua River, Northeast China	Riparian population	Fish	0.648 (0.16–199)	[83]
Songhua River, Northeast China	Fishermen	Fish	2.25 (0.02–18.1)	[89]
Wanshan Hg mining area, China	Local residents	Rice	2.8 (0.8–5.6)*	[13]
			1.3 (0.2–3.6)*	
			1.5 (0.5–3.5)*	
Wuchuan Hg mining area,China	Local residents	Rice	1.38 (0.45–5.89)*	[14]
Tongren Hg mining area, China	Local residents	Rice	4.26 (1.87–10.6)*	[90]
			4.55 (2.29–9.55)*	

MH = Maternal hair;

* MeHg.

**Table 4.** The comparison of Se concentrations in rice in China/μg·g^−1^.

**Location**	***n***	**Mean ± S.D.**	**Range**	**Ref.**
Jilin	70	0.008		[107]
Jiangsu	52	0.035 ± 0.009	0.015–0.056	[108]
Eleven Provinces	30	0.025 ± 0.011	0.011–0.055	[108]
Whole China	91	0.029 ± 0.019	0.004–0.111	[109]
Nanjing, Jiangsu	34	0.039 ± 0.029	0.002–0.202	[110]
Jiangxi, Hunan, Anhui, and Guangdong	70	0.028 ± 0.013	0.003–0.075	[111]
Guangzhou, Guangdong	25	0.058 ± 0.030	0.011–0.125	[112]
Taiyuan, Shanxi	17	0.037	0.015–0.062	[113]
Wanshan, Guizhou	6		0.4–1.06	[11]
Qingzhen, Guizhou	4		0.24–1.01	[11]
Whole China	523	0.088	0.002–1.37	[106]
